# Clinical and echocardiographic features of children with rheumatic heart disease and their serum cytokine profile

**Published:** 2012-10-19

**Authors:** Sulafa Khalid Mohamed Ali, Inaam Noor Eldaim, Samia Hassan Osman, Sahar Mohamed Bakhite

**Affiliations:** 1 Jafaar Ibn Ouf Children’s Hospital, Sudan; 2 Department of Paediatrics and Child Health, Faculty of Medicine, University of Khartoum, Sudan; 3 Institute of Endemic Diseases, University of Khartoum, Sudan

**Keywords:** Rheumatic heart disease, serum cytokine profile, valvular lesions, children

## Abstract

Acute rheumatic fever (ARF) and rheumatic heart disease (RHD) constitute important public health problems in developing countries. Children with ARF and RHD seen at Children’s Hospital-Sudan from May 2008-2009 were examined clinically and by echocardiography. Blood cytokines (interleukin 10 (IL10), Tumor necrosis factor alpha (TNF- alpha) and interferon gamma (IFN-gamma) were done. Thirty six children were enrolled; 63% had established RHD, and 37% ARF. Mitral regurgitation (MR) was the most common lesion (94%).Ninety five percent of the valve lesions were severe. The serum interleukin IL10 level ranged between 3-6 pg/ml. TNF alpha levels were 9- 100 pg/ml in 12 patients (40%), 101-1000 pg/ml in 10 patients (33%), more than 1000 in 8 patients (26%). The level of IFN gamma ranged between 2-7 pg/m in all patients except 2 (84 and 135 pg/ml). RHD is manifested with severe valvular lesions and a high TNF alpha indicating and ongoing inflammation.

## To the editors of the Pan African Medical Journal

Rheumatic heart disease (RHD) is a devastating squeal of acute rheumatic fever (ARF) in Africa. Recent evidence suggests that T-cell lymphocytes play an important role in the pathogenesis of rheumatic carditis. CD4+ T cells are most likely the ultimate effectors of chronic valve lesions in RHD [1].

A prospective cross sectional study was carried at the Children’s hospital from May 2008-2009 after obtaining ethical approval. All patients with ARF carditis and /or established RHD were enrolled. Patients were examined clinically and by echocardiography (echo).

### Echocardiography

Criteria for valve dysfunction were applied using standard reference values published by the American and European Societies of Echo [2]. The maximum anteroposterior diamaeter of the anterior mitral leaflet (AML) and posterior mitral leaflet (PML) tip thickness were measured from the parastenrnal long axis view in diastole when the valve is fully open.

### Cytokine Measurement

IFN-gamma (T-Helper 1), IL-10(T-Helper 2), TNF-alpha cytokines were measured using cytokine specific ELISA. The results were compared with published standard controls form healthy children. Reference values for children (pg/ml): IFN gamma: 4-6 (3.3-7.8), TNF alpha: 2-3 (1.0-3.1), IL-10: 2-4 (1.3-9.9) [3].

### Clinical Features

Twenty three patients (63%) had established RHD, 13 patients had ARF (37%), of these, only 1 patient had the first episode of ARF. Clinical features are summarized in Table 1.

**Table 1:** Clinical Features of Patients with acute rheumatic fever/Rheumatic heart disease

Feature	No	%
Age (Years)		
5-7	5	14
7-10	5	14
10-16	26	72
Sex (Female/male)	1.2:1	
Fever	21	58
Heart failure	34	94
Arthritis	7	19
Skin rash	1	2.8
Skin nodules	1	2.8
Chorea	1	2.8

### Echo Findings

**Figure 1:**
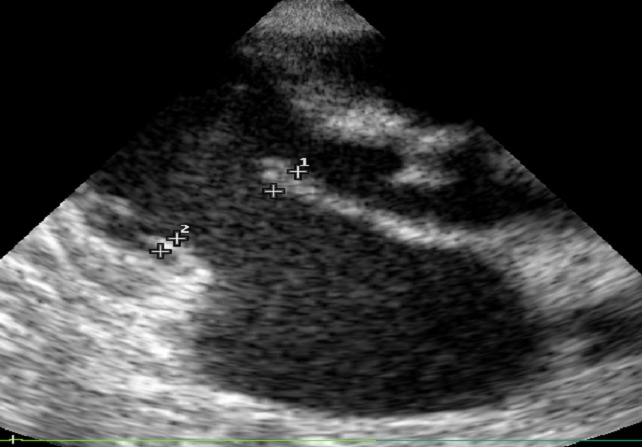
Parasternal long axis echocardiographic view showing the measurement of the anterior (AML) and posterior (PML) mitral leaflets

Echo features are summarized in Table 2. The AML thickness ranged between 4.2-11.8 mm/m^2^ with a mean of 6.3 mm/m^2^, PML thickness ranged from 3.7-9 with a mean of 5.7 mm/m^2^ (Figure 1).

**Table:** Echocardiographic features of patients with acute rheumatic fever/Rheumatic heart disease

Lesion	No	%
Isolated MR	18	50
Mild-Moderate	2	12
Severe	16	88
Isolated AR (severe)	2	5
Combined MR and AR (severe)	14	38
MS (severe)	1	2.8
Low Ejection Fraction	9	25
Pulmonary hypertension	22	61

MR: Mitral regurgitation; AR: Atrial regurgitation; MS: Mitral Stenosis

### Cytokine Levels

Cytokine levels are shown in Table 3. Results were available for 30 patients. There was no correlation between the level of cytokines and the severity of valve lesions.

**Table 3:** Serum Cytokine levels in patients with ARF/RHD

Patient	IL10	TNF-alpha	IFN-gamma
1.	13.8	10	6
2.	5	9	5
3.	4	9	3
4.	3	18	4
5.	4	9	5
6.	3	1085	5
7.	3	587	7
8.	5	176	6
9.	5	4.8	3
10.	6	15	4
11.	3	12	5
12.	4	520	6
13.	3	1918.8	84.6
14.	3	15	6
15.	4	130.5	5
16.	5	46.7	6
17.	5	76	6
18.	6	344	5
19.	4	645.6	135
20.	5	243.6	4
21.	4	180.7	3
22.	5	151.4	4
23.	93.2	436.2	2
24.	4	18	5
25.	3	2898.8	4
26.	4	1504.2	3
27.	5	1474.8	2
28.	4	1022.5	3
29.	5	272.9	2
30.	4	570.2	6

The frequency of heart failure as well as the degree of valve dysfunction were strikingly more severe than other parts of the world. Reports from Sudan as well as from India had found that the mitral valve was the most affected valve, however the second most common valve dysfunction in Indian children was combined MR (Mitral regurgitation)/MS (Mitral stenosis) in contrast to our patients who had MR/AR (Atrial regurgitation) as the second most common valve dysfunction. Many patients presented with established RHD with no appreciation of the first symptoms of ARF, the so called “indolent carditis” [4–6]. These findings strongly undervalue the benefit of secondary prophylaxis as the patients presented with valves that are already damaged. Families of such patients are not expected to adhere to penicillin prophylaxis even if they were picked up early.

Leaflet thickening is a constant sign of RHD that can easily detected by echo, however there are few reference values for this measurements in the literature [7]. In a recent study, Bo et al measured the absolute thickness of AML in patients with RHD, however they did not index it to the patients’ surface area [8]. In a previous study we measured MV leaflet thickness in normal children and found that the mean was 2.8 mm/m^2^ (+/- 0.2mm) for the AML and 2.0 mm/m^2^ (+/- 0.1mm) for the PML (Sulafa KM Ali, unpublished data). The current study revealed that the MV leaflets in patients with RHD are significantly thickened compared to normal. We encourage echocardiographers to use these reference values when reporting mitral valve thickening in order to avoid subjective errors.

In this study the levels of cytokines indicate increased TNF alpha and low levels of IFN gamma and IL10. Similar results were found by Chen et al in patients with rheumatic MS [9]. It was shown that TNF- alpha was increased in patients with ARF as well as those with RHD, in accordance to our results, this indicate that there is an on-going inflammatory activity in these patients and raises the question of the role of immune modulative drugs in these patients.. In the latter study there was a positive correlation between the level of TNF-alpha and the severity of mitral valve dysfunction as well as that of heart failure. In addition, Mohamed et al from Egypt found a high level of TNF-alpha as well as 238G/A and -308G/A polymorphisms in patients with RHD correlating with a more severe outcome of RHD, findings that may explain the severe nature of RHD in our patients [10].

In our patients, the statistical correlation between the severity of RHD and the level of cytokines was not possible due to the fact that almost all patients had severe valve dysfunction and high level of TNF-alpha.

RHD is manifested with high rates of recurrence of ARF and severe valvular lesions with dominant MR, high prevalence of poor myocardial function and pulmonary hypertension. Most patients had a high TNF alpha which indicates an ongoing inflammation.
